# Editorial for Special Issue: Gel Films and Coatings Applied in Active Food Packaging

**DOI:** 10.3390/gels9090743

**Published:** 2023-09-13

**Authors:** Aris E. Giannakas

**Affiliations:** Department of Food Science and Technology, University of Patras, 30100 Agrinio, Greece; agiannakas@upatras.gr

## 1. Introduction

Nowadays, the global trends of bioeconomy and sustainability require the use of biobased raw materials in all scientific and application fields to reduce the global carbon dioxide fingerprint. In this direction, natural biopolymers such as chitosan, sodium alginate, gelatin, pectin, and xanthan are potential candidates for replacing polymers derived from mineral oils in new food packaging applications. At the same time, nanotechnology provides innovative applications in the field of food packaging, similar to those provided in the biomedical and pharmaceutical fields. By incorporating biobased bioactive compounds such as essential oils, derivatives of essential oils, propolis, anthocyanin, aloe vera, acacia gum, and collagen in biopolymers, novel gels and hydrogels can be developed with the control release properties of enriched bioactive compounds, which can be applied as smart films and coatings in the food industry. Moreover, nanomaterials such as nanoclays and natural zeolites can be used as new, cheap, and natural abundant nanocarriers for such bioactive compounds and enhance their release control properties from the biopolymer matrix. These bioactive films and coating materials enhance solubility, improve bioavailability, facilitate controlled release, and protect bioactive ingredients during manufacture and storage. The current Special Issue provides a fresh bouquet of articles on the bioactive gel films and coatings applied to active food packaging.

## 2. Contributions

In the article of Bhatia et al. [1], hydrogel-based films loaded with varying concentrations of Melissa officinalis (MOEO) (0.1%, 0.15%, and 0.2%) were prepared using the solvent-casting method, and their physicochemical and antioxidant properties were examined. The incorporation of MOEO into the films gave films with a higher elongation at break (EAB) (30.24–36.29%), lower tensile strength (TS) (3.48–1.25 MPa), lower transparency (87.30–82.80%), higher water barrier properties, and higher antioxidant properties, indicating that MOEO has the potential to be used in active food packaging material for potential applications.

The same research group developed sodium alginate (SA) and acacia gum (AG) hydrogel-based films loaded with cinnamon essential oil (CEO) and revealed that, as the oil concentration in the films increased, the thickness and elongation at break (EAB) increased, while the transparency, tensile strength (TS), water vapor permeability (WVP), and moisture content (MC) decreased. As the concentration of CEO increased, the hydrogel-based films demonstrated a significant improvement in their antioxidant properties [2]. Overall, it was concluded that the incorporation of CEO into edible SA–AG composite films presented a promising strategy for the production of hydrogel-based films with the potential to serve as food-packaging materials.

Elsebaie et al. [3] studied the preparation, characterization (physical, mechanical, optical, and morphological properties, as well as antioxidant and antimicrobial activities), and packaging application of chitosan (CH)-based gel films containing varying empty green pea pod extract (EPPE) concentrations (0, 1, 3, and 5% *w*/*w*). Their studies revealed that adding EPPE to CH increased the thickness (from 0.132 ± 0.08 to 0.216 ± 0.08 mm), density (from 1.13 ± 0.02 to 1.94 ± 0.02 g/cm^3^), and opacity (from 0.71 ± 0.02 to 1.23 ± 0.04), while decreasing the water vapor permeability, water solubility, oil absorption ratio, and whiteness index from 2.34 to 1.08 × 10^−10^ g^−1^ s^−1^ pa^−1^, from 29.40 ± 1.23 to 18.75 ± 1.94%, from 0.31 ± 0.006 to 0.08 ± 0.001%, and from 88.10 ± 0.43 to 77.53 ± 0.48, respectively. The EPPE films had a better tensile strength (maximum of 26.87 ± 1.38 MPa), elongation percentage (maximum of 58.64 ± 3.00%), biodegradability (maximum of 48.61% after 3 weeks), and migration percentages than the pure CH gel film. By adding EPPE, the antioxidant and antibacterial activities of the film were improved. Furthermore, compared to control samples, corn oil packaging in CH-based EPPE gel films slowed increases in thiobarbituric acid and superoxide. As an industrial application, CH-based EPPE films have the potential to be beneficial in food packaging.

Salmas et al. [4] presented a novel adsorption process for producing a thymol-halloysite nanohybrid and dispersed it in a chitosan/poly-vinyl-alcohol matrix. The results indicated that the increased fraction of thymol from thyme oil significantly enhanced the antimicrobial and antioxidant activities of the prepared chitosan-poly-vinyl-alcohol gel. The use of halloysite increased the mechanical and water–oxygen barrier properties, leading to a controlled release process of thymol, which extended the preservation and shelf-life of kiwi fruits. Finally, the results indicated that the halloysite improved the properties of the chitosan/poly-vinyl-alcohol films, and the thymol made them further advantageous.

The same research group [5] developed a green method for producing natural zeolite (TO@NZ) nanostructure rich in thymol. This material was used to prepare sodium-alginate/glycerol/xTO@NZ (ALG/G/TO@NZ) nanocomposite active films for the packaging of soft cheese to extend its shelf-life. The water vapor transmission rate, oxygen permeation analyzer, tensile measurements, antioxidant measurements, and antimicrobial measurements were used to estimate the film’s water and oxygen barrier and mechanical properties and the nanostructure’s nanoreinforcement activity, antioxidant activity, and antimicrobial activity. The findings from this study revealed that the ALG/G/TO@NZ nanocomposite film could be used as an active packaging film for foods with enhanced mechanical properties, oxygen and water barrier, and antioxidant and antimicrobial activities, and it is capable of extending food shelf life.

Puscaselu et al. [6] developed biopolymer-based films (agar and sodium alginate) and tested their properties following additions of 7.5% and 15% (*w*/*v*) of various essential oils (lemon, orange, grapefruit, cinnamon, clove, chamomile, ginger, eucalyptus, or mint). The samples were tested immediately after their development and after one year of storage to examine the possible long-term property changes. All the films showed reductions in mass, thickness, and microstructure, as well as their mechanical properties. The most considerable variations in their physical properties were observed in the 7.5% lemon oil sample and 15% grapefruit oil sample, with the largest reductions in mass (23.13%), thickness (from 109.67 µm to 81.67 µm), and density (from 0.75 g/cm^3^ to 0.43 g/cm^3^). However, the microstructures of the samples were considerably improved. Although the addition of lemon essential oil prevented a reduction in mass during the storage period, it favored the degradation of the microstructure and a loss of elasticity (from 16.7% to 1.51% for the sample with 7.5% lemon EO and from 18.28% to 1.91% for the sample with 15% lemon EO). Although the addition of the essential oils of mint and ginger resulted in films with a more homogeneous microstructure, the increase in concentration favored the appearance of pores and modifications in the color parameters. Apart from the films with added orange, cinnamon, and clove EOs, the antioxidant capacity of the films decreased during storage. The most obvious variations were identified in the samples with lemon, mint, and clove EOs. The most unstable samples were those with added ginger (95.01%), lemon (92%), and mint (90.22%). The same research group [7] furthered their research on the development of such films and tested the modification of the properties of the previously developed biopolymeric materials, by adding 10 and 20% *w*/*v* essential oils, respectively, of lemon, grapefruit, orange, cinnamon, clove, mint, ginger, eucalypt, and chamomile. Films with a thickness between 53 and 102 µm were obtained, with a roughness ranging between 147 and 366 nm. Most films had a water activity index significantly below what is required for microorganism growth, as low as 0.27, while all the essential oils induced a microbial growth reduction or 100% inhibition. Tested for an evaluation of their physical, optical, microbiological, or solubility properties, all the films with an addition of essential oil to their composition showed improved properties compared to the control sample.

Chit et al. [8] developed a single-layer coating using chitosan (Ch) and sodium alginate (SA) solutions, and their gel coating (ChCSA) was formed via layer-by-layer (LbL) electrostatic deposition using calcium chloride (C) as a cross-linking agent to improve the storage qualities and shelf-life of fresh-cut purple-flesh sweet potatoes (PFSP). The preservative effects of the single-layer coating in comparison to LbL on the quality parameters of the fresh-cut PFSPs, including color change, weight loss, firmness, microbial analysis, CO_2_ production, pH, solid content, total anthocyanin content (TAC), and total phenolic content (TPC), were evaluated during 16 days of storage at 5 °C. Uncoated samples were applicable as a control. The result established the effectiveness of coating in reducing microbial proliferation (~2 times), color changes (~3 times), and weight loss (~4 times), with negligible firmness losses after the storage period. In addition, the TAC and TPC were better retained in the coated samples than in the uncoated samples. In contrast, quality deterioration was observed in the uncoated fresh cuts, which progressed with storage time. Relatively, gel coating ChCSA showed superior effects on preserving the quality of the fresh-cut PFSPs and could be suggested as a commercial method for preserving fresh-cut purple-flesh sweet potato and other similar roots.

Al Hilifi et al. [9] developed aloe vera gel (AVG)-based edible coatings enriched with anthocyanin and investigated the effect of different formulations of these aloe-vera-based edible coatings, such as neat AVG (T1), AVG with glycerol (T2), aloe vera with 0.2% anthocyanin + glycerol (T3), and AVG with 0.5% anthocyanin + glycerol (T4), on the postharvest quality of fig (Ficus carica L.) fruits under refrigerated conditions (4 °C) for up to 12 days of storage with 2-day examination intervals. The results of the present study revealed that the T4 treatment was the most effective for reducing the weight loss in the fig fruits throughout the storage period (~4%), followed by T3, T2, and T1. The minimum weight loss after 12 days of storage (3.76%) was recorded for the T4 treatment, followed by T3 (4.34%), which was significantly higher than that of uncoated fruit (~11%). The best quality attributes, such as the total soluble solids (TSS), titratable acidity (TA), and pH, were also demonstrated in the T3 and T4 treatments. The T4 coating caused a marginal change of 0.16 in the fruit titratable acidity, compared to the change of 0.33 in the untreated fruit control after 12 days of storage at 4 °C. Similarly, the total soluble solids in the T4-coated fruits increased marginally (0.43 °Brix) compared to that in the uncoated control fruits (>2 °Brix) after 12 days of storage at 4 °C. The results revealed that the incorporation of anthocyanin content into AVG is a promising technology for the development of active edible coatings to extend the shelf life of fig fruits.

Sheikha et al. [10] studied the effect of a xanthan coating containing various concentrations (0, 1, and 2%; *w*/*v*) of ethanolic extract of propolis (EEP) on the physicochemical, microbial, and sensory quality indices in mackerel fillets stored at 2 °C for 20 days. The pH, peroxide value, K-value, TVB-N, TBARS, microbiological, and sensory characteristics were determined every 5 days over the storage period (20 days). The samples treated with xanthan (XAN) coatings containing 1 and 2% of EEP were shown to have the highest level of physicochemical protection and maximum level of microbial inhibition (*p* < 0.05) compared to the uncoated samples (control) over the storage period. Furthermore, the addition of EEP to XAN was more effective in notably preserving (*p* < 0.05) the taste and odor of the coated samples compared to the controls.

## 3. Conclusions

In conclusion, we are thankful to have edited this Special Issue, as it collects relevant contributions that reflect the increasingly widespread interest in gel films and coatings applied to food packaging. Overall, it was revealed that such films and gel coatings can be used to extend the shelf life of various food products such as fruits, dairy products, and fish products. We look forward to this Special Issue reaching the highest scientific audience and promoting the second edition.
